# The ketone body β-hydroxybutyrate alleviates CoCrMo alloy particles induced osteolysis by regulating NLRP3 inflammasome and osteoclast differentiation

**DOI:** 10.1186/s12951-022-01320-0

**Published:** 2022-03-09

**Authors:** Yanglin Wu, Yun Teng, Chenhui Zhang, Ying Pan, Qin Zhang, Xu Zhu, Naicheng Liu, Xinlin Su, Jun Lin

**Affiliations:** 1 Department of Orthopaedics, The First Affiliated Hospital of Soochow University, Soochow University, No. 188 Shizi Street, Suzhou, 215006 Jiangsu China; 2 Orthopaedic Institute, Medical College, Soochow University, Suzhou, 215007 China; 3 Department of Infectious Diseases, The Second Affiliated Hospital, Zhejiang University School of Medicine, Hangzhou, China

**Keywords:** β-hydroxybutyrate, NLRP3 inflammasome, Osteoclast, Osteolysis, Wear particles

## Abstract

**Background:**

Aseptic Loosening (AL) following periprosthetic osteolysis is the main long-term complication after total joint arthroplasty (TJA). However, there is rare effective treatment except for revision surgery, which is costly and painful to the patients. In recent years, the ketone body β-hydroxybutyrate (BHB) has attracted much attention and has been proved to be beneficial in many chronic diseases. With respect to the studies on the ketone body β-hydroxybutyrate (BHB), its anti-inflammatory ability has been widely investigated. Although the ketone body β-hydroxybutyrate has been applied in many inflammatory diseases and has achieved considerable therapeutic efficacy, its effect on wear particles induced osteolysis is still unknown.

**Results:**

In this work, we confirmed that the anti-inflammatory action of β-hydroxybutyrate (BHB) could be reappeared in CoCrMo alloy particles induced osteolysis. Mechanistically, the ketone body β-hydroxybutyrate (BHB) deactivated the activation of NLRP3 inflammasome triggered by CoCrMo alloy particles. Of note, this inhibitory action was independent of Gpr109a receptor as well as histone deacetylase (HDAC) suppression. Furthermore, given that butyrate, one kind of short chain fatty acid (SCFA) structurally related to β-hydroxybutyrate (BHB), has been reported to be an inhibitor of osteoclast, thus we also investigate the effect of β-hydroxybutyrate (BHB) on osteoclast, which was contributed to bone resorption. It was found that β-hydroxybutyrate (BHB) did not only affect osteoclast differentiation, but also inhibit its function. Unlike the inflammasome, the effect of β-hydroxybutyrate (BHB) on osteoclast may mainly rely on histone deacetylase (HDAC) suppression.

**Conclusions:**

In general, our study showed that the alleviation of osteolysis may owe to the effect of β-hydroxybutyrate (BHB) on inflammasome deactivation and osteoclast.

**Graphical Abstract:**

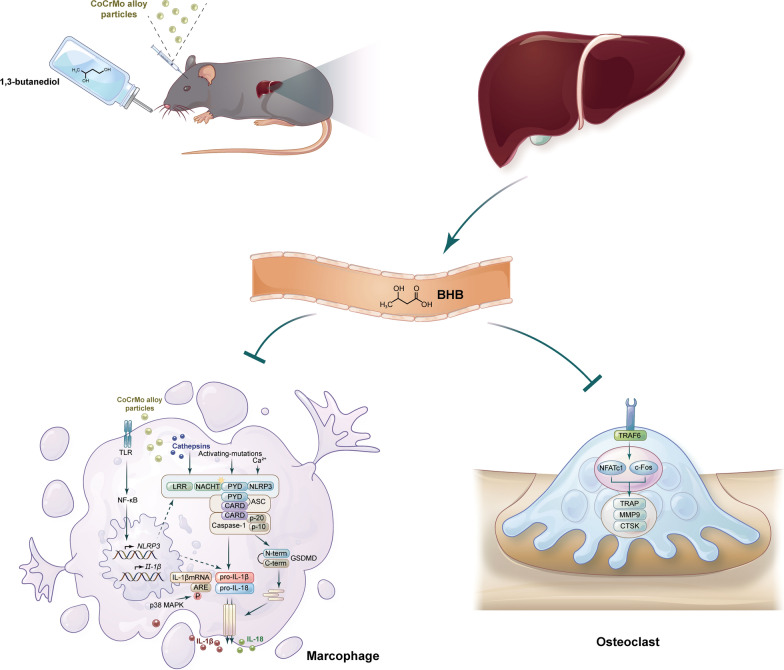

**Supplementary Information:**

The online version contains supplementary material available at 10.1186/s12951-022-01320-0.

## Background

Total joint arthroplasty (TJA), as the most effective treatment for end-stage arthritis, provide reliable long-term improvements in the aspects of patients’ joint function, pain as well as life quality [1]. However, it was reported that 10–70% of patients had suffered aseptic loosening (AL) following periprosthetic osteolysis after TJA [2]. Unfortunately, patients underwent TJA accounts for more than 40% under the age of 65, and the proportion of patients in this age group is gradually increasing [3]. As young age is one of the most known risk factors for osteolysis, the incidence rate of aseptic loosening will undoubtedly increase in a steady way [3]. Finally, these patients with aseptic loosening have to face revision surgery, a costly and painful process. Periprosthetic osteolysis has become a growing public health problem worldwide over time.

Recently studies have confirmed that the activation of NLRP3 inflammasome contributes to the pathogenesis of osteolysis induced by implant wear particles [3–7]. The NLRP3 inflammasome, a cytosolic multimeric protein complex, is composed of the sensor protein Nlrp3 (Nod-like receptor pyrin domain 3), the adaptor protein ASC (apoptosis-associated speck-like protein containing a CARD), and caspase-1, which controls the cleavage of caspase-1 and the secretion of IL-1β and IL-18 upon pathological stimulations in macrophages [8]. It is a cellular sensor of harmful situations that is activated by diverse damage-associated molecular patterns (DAMPs) such as ATP, toxins, ceramides, excess glucose, cholesterol crystals, amyloids, and urate [9–15]. The specific pathological process of osteolysis is that wear particles are phagocytosed by circulating macrophages, which in turn activate the NLRP3 inflammasome due to lysosomal rupture and leakage of the protease Cathepsin B into the cytoplasm, thus inducing the process of pro-IL-1β and secretion of mature, cleaved IL-1β [4, 7]. As the main pathogenic factor, the inflammatory cytokine of IL-1β is considered to increase the recruitment and differentiation of osteoclast precursors, which was attributed to the bone resorption [16].

The ketogenic diet has attracted more and more attention because the ketone body may be a metabolic mediator for the benefits of calorie restriction [17, 18]. The ketogenic diet is a high fat, low carbohydrate with moderate protein diet [18]. It has been used in multiple diseases, such as epilepsy, obesity, type2 diabetes, and so on [19–26]. Recent studies showed that the benefits of ketogenic diet were mostly due to the ability to regulate inflammation by the ketone body β-hydroxybutyrate (BHB) [18, 27–30]. For instance, Kim et al. [27] reported that β-hydroxybutyrate (BHB) modulated NLRP3 inflammasome activity in diabetes with the cardiovascular disease while Goldberg et al. [28] demonstrated that β-hydroxybutyrate (BHB) deactivates neutrophil NLRP3 inflammasome to relieve gout flares [27, 28]. More recently, Youm et al. showed β-hydroxybutyrate (BHB) blocked the NLRP3 inflammasome activation upon urate crystals and lipotoxic fatty acids to alleviate the corresponding diseases [31]. All these studies suggested that the ability of β-hydroxybutyrate (BHB) in regulating inflammation is mainly due to the deactivation of inflammasome. However, the impact of β-hydroxybutyrate (BHB) on CoCrMo alloy particles induced inflammasome activation is still unknown.

In the present study, we reported that β-hydroxybutyrate (BHB) rather than another ketone body acetoacetate (AcAc) could inhibit the NLRP3 inflammasome activation induced by CoCrMo alloy particles. Furthermore, we also found that β-hydroxybutyrate (BHB) did not only regulate inflammation, but also played an important role in the differentiation and function of osteoclasts. More importantly, both R-BHB and its chiral compound enantiomer S-BHB have the same inhibitory effect on inflammasome activation and osteoclast differentiation. Our work showed that the anti-osteolysis effects of β-hydroxybutyrate (BHB) may be attributed to the inhibition of osteoclast differentiation and the NLRP3 inflammasome.

## Results

### The characteristic of CoCrMo alloy particles

**Fig. 1:**
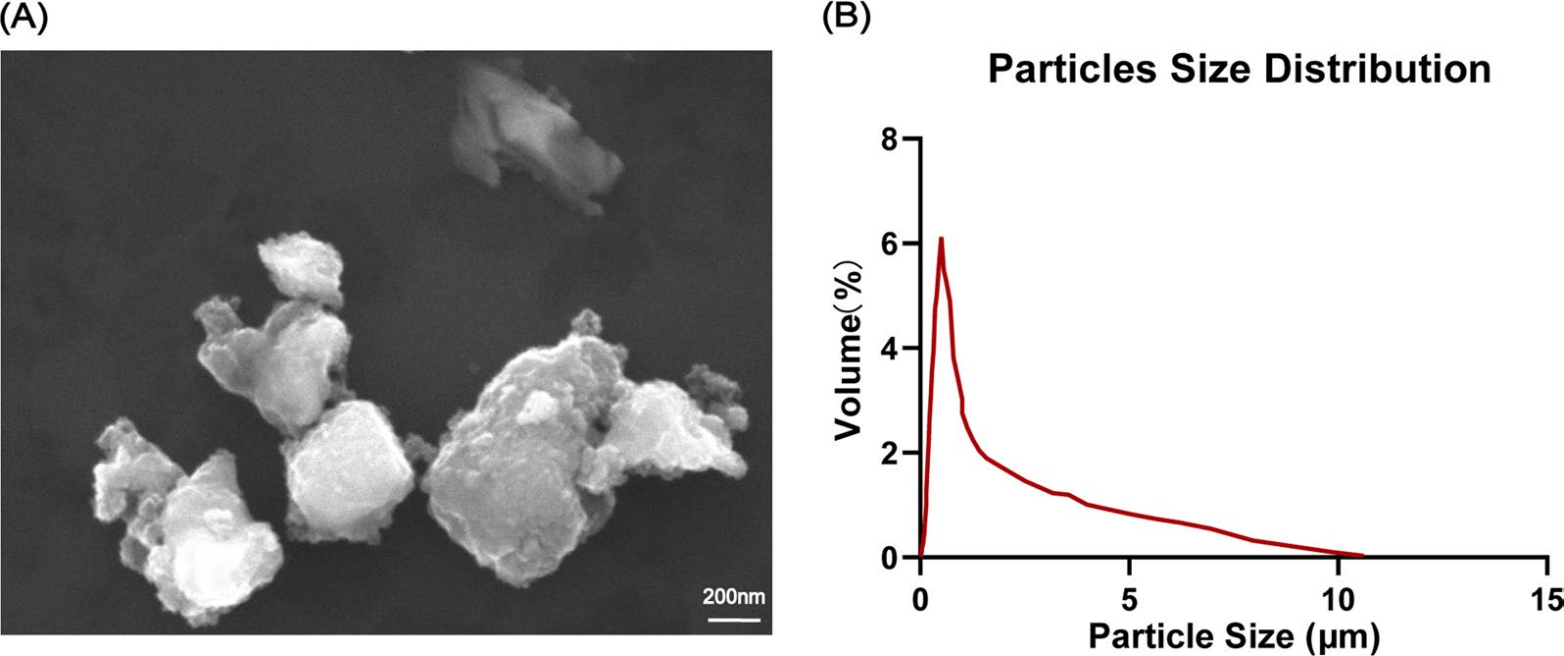
The properties of CoCrMo alloy particles. **A** Representative scanning electron microscopy (SEM) image of CoCrMo alloy particles. Scale bar: 200 nm. **B** The size distribution of CoCrMo alloy particles

CoCrMo alloys have become the first choice for artificial joints in the past 60 years because of their excellent mechanical properties, wear resistance, corrosion resistance, and good biocompatibility [32]. In this study, a kind of irregular shaped CoCrMo alloy particles was applied for in vivo and in vitro studies (Fig. 1A). The average particles size was 1.69 μm (range from 0.02–10.6 μm, medium 0.564 μm) (Fig. 1B). CoCrMo alloy particles of this range have been reported that it can be phagocytosed by macrophages and lead to a strong inflammation response [5]. It was also identified around periprosthetic tissues [33, 34].

### β-hydroxybutyrate suppress NLRP3 inflammasome activation caused by wear particle

**Fig. 2:**
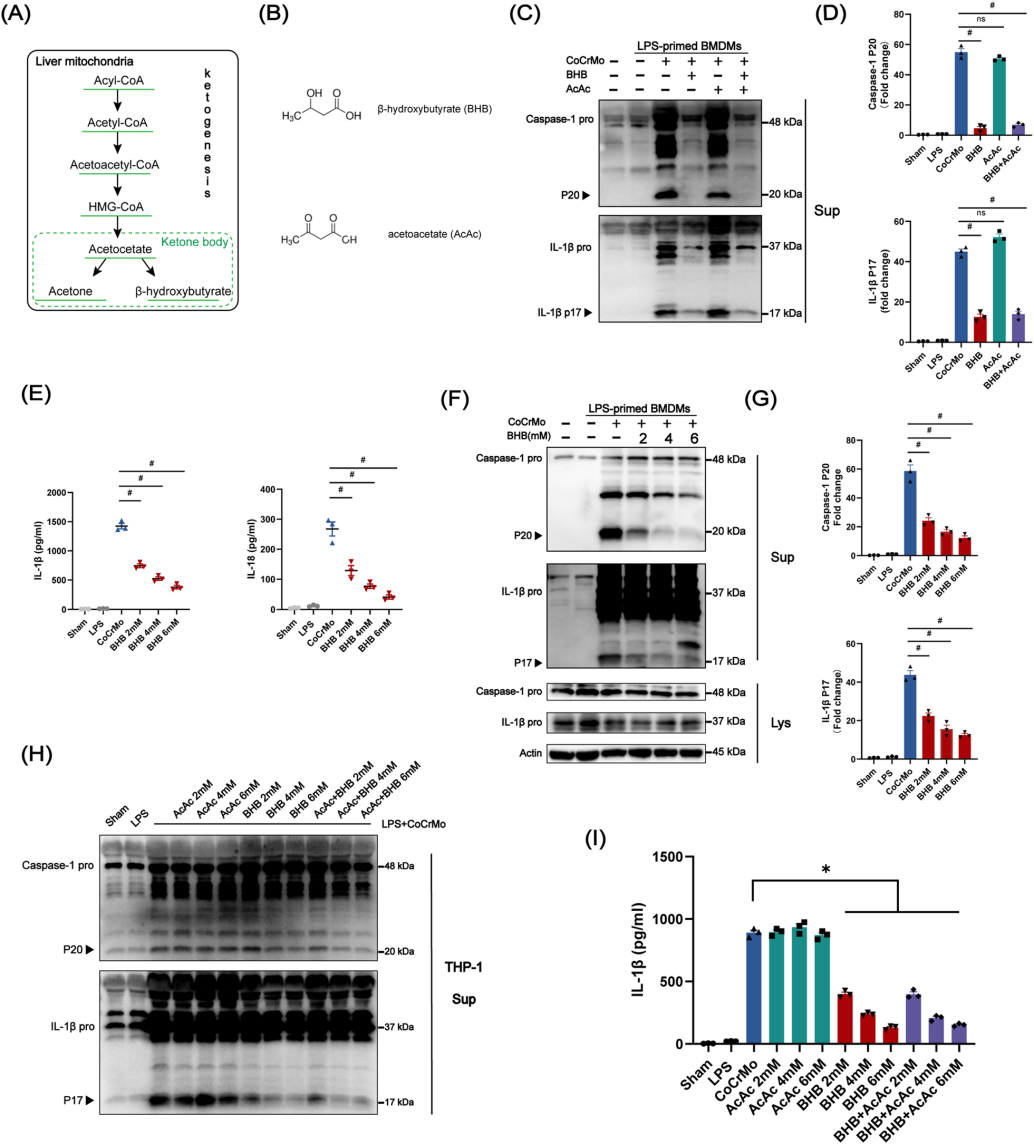
BHB inhibits NLRP3 inflammasome activation induced by CoCrMo particles. **A** Ketogenesis in hepatic mitochondria. **B** Structure of β-hydroxybutyrate (BHB) and acetoacetate (AcAc). **C** BMDMs (LPS-primed) treated with BHB (2 mM), AcAc (2 mM), or BHB (2 mM) + AcAc (2 mM) and stimulated with CoCrMo particles. Supernatants were collected for Caspase-1 and IL-1β detection by western blot. **D** Quantification of active Caspase-1 (P20) and active IL-1β (P17). **E**–**G** BMDMs (LPS-primed) treated with different concentrations of BHB and CoCrMo alloy particles. **E** Supernatants were collected for IL-1β and IL-18 analysis by ELISA. **F** The cell lysates and supernatants were detected for Caspase-1 and IL-1β by western blot. **G** Quantification of active Caspase-1 (P20) and active IL-1β (P17). **H** LPS-primed THP-1 macrophages (PMA-differentiated) treated with different concentrations of BHB, AcAc or BHB + AcAc and stimulated with CoCrMo particles. Supernatants were collected for Caspase-1 and IL-1β detection by western blot. **I** Supernatants were collected for IL-1β analysis by ELISA. n = 3. Results are mean ± SEM. *p < .05, ^#^p < .001

The ketone body is produced by ketogenesis within hepatic mitochondria (Fig. 2A). It is composed of acetone, acetoacetate (AcAc), and β-hydroxybutyrate (BHB) (Fig. 2B) [35]. Researches of β-hydroxybutyrate (BHB) on the NLPR3 inflammasome activation has covered many areas, but there is on study about the effect of β-hydroxybutyrate (BHB) on inflammasome activation induced by wear particles. To verify whether the ketone body affects the inflammasome activation induced by wear particles, we treated LPS (lipopolysaccharide)-primed BMDMs (bone marrow–derived macrophages) with CoCrMo alloy particles along with β-hydroxybutyrate (BHB) or acetoacetate (AcAc) and detected the cleaved caspase-1 (P20) and IL-1β (P17). It is suggested that BHB rather than AcAc inhibited both the CoCrMo particles-induced caspase-1 cleavage into active form P20 and the process of biologically active IL-1β, P17 (Fig. 2C and D). We found that the inhibitory effect of BHB on inflammasome activation, IL-1β, and IL-18 secretion was in a dose-dependent manner (Fig. 2E–G). Then we also confirmed the inhibitory action was mediated by BHB rather than AcAc in THP-1 (Tohoku Hospital Pediatrics-1) macrophages (PMA-differentiated) (Fig. 2H-I, Additional file 1: Fig. S1). Collectively, our work demonstrated that BHB has an effective suppression on inflammasome activation induced by CoCrMo particles.

### BHB interferes with ASC assembly and represses pyroptosis

**Fig. 3:**
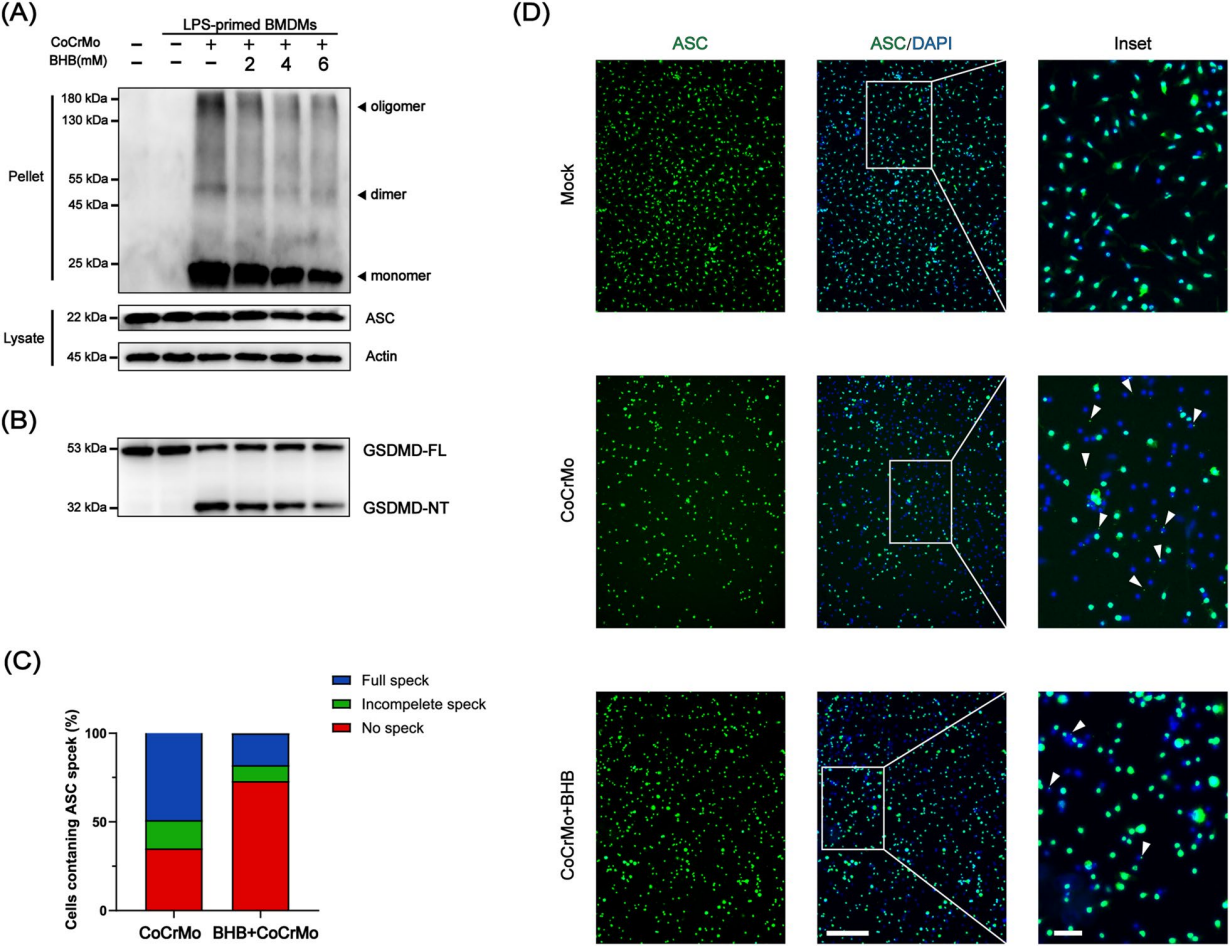
BHB inhibited ASC oligomerization, speck formation, and pyroptosis. **A** Representative image of Cross-linked cytosolic pellets of ASC oligomerization by western blot. **B** Representative western blot image of GSDMD in cell lysates. GSDMD NT: Gasdermin-D N-terminal. GSDMD FL: Gasdermin-D full length. **C** Quantification analysis of ASC speck. **D** Representative images of ASC speck formation by immunofluorescence. Scale bar: 200 μm (panel), 20 μm (inset). White arrows indicate ASC specks

ASC, NLRP3 dependent, nucleation-induced oligomerization is considered to be a common mechanism of NLRP3 inflammasome activation [8]. We found that the active inflammasome complex formation formed by ASC protein oligomerization with NLRP3 and pro-caspase-1 was dose-dependently diminished by BHB (Fig. 3A). Furthermore, BHB could also restrict ASC speck formation activated by CoCrMo particles (Fig. 3C and D). Thus, BHB inhibited ASC oligomerization, assembly, and speck formation to control the NLRP3 inflammasome activation.

Pyroptosis, a type of inflammatory cell death, occurs after the activation of inflammasome. To confirm the possibility that the inhibition of IL-1β secretion by BHB is attributed to pyroptosis suppression. The GSDMD-NT (Gasdermin-D N-terminal) fragment, which is generated by active caspase-1 cleavage, is detected by western blot in cell lysate. We observed BHB dose-dependently inhibited the GSDMD-NT generation compared with CoCrMo particles treated alone (Fig. 3B). To confirm the inhibition of BHB on pyroptosis, Calcein and propidium iodide (PI) staining, a method of cell death detection, also confirmed the inhibitory effect of BHB (Additional file 1: Fig. S2). Furthermore, we measured the release of Lactate Dehydrogenase (LDH). It is suggested that BHB dose-dependently suppressed CoCrMo alloy particles induced LHD release (Additional file 1: Fig. S3).

### The effect of BHB on inflammasome does not dependent on Gpr109a receptor or inhibiting histone deacetylase

**Fig. 4:**
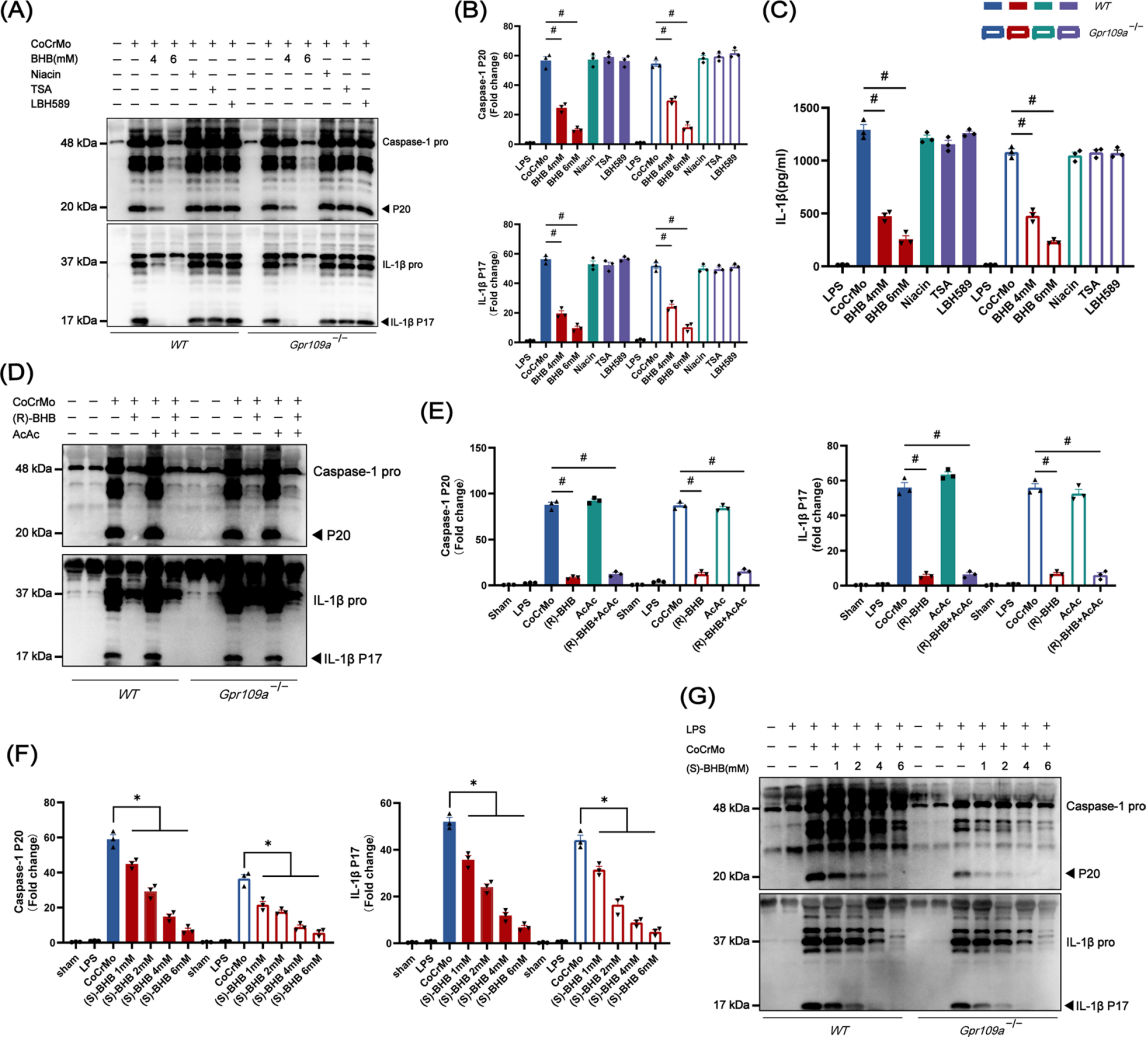
The suppression of BHB on NLRP3 inflammasome activation was independent of the Gpr109a receptor and histone deacetylase suppression. **A**–**C** BMDMs (LPS-primed) were treated with different doses of BHB, Niacin (100 μM), TSA (50 nM), LBH589 (25 nM) and stimulated with CoCrMo particles. **A** Supernatants were collected for Caspase-1 and IL-1β detection by western blot. **B** Quantification of active Caspase-1 (P20) and active IL-1β (P17). **C** Analysis of IL-1β by ELISA in supernatants. **D**–**E** BMDMs (LPS-primed) from wild type (WT) or Gpr109a deficient mice were treated with (R)-BHB (4 mM), AcAc (4 mM), and (R)-BHB (4 mM) + AcAc (4 mM) and stimulated with CoCrMo particles. **D** Representative image of Caspase-1 and IL-1β in supernatants by western blot. **E** Quantification of active Caspase-1 (P20) and active IL-1β (P17). **F**–**G** BMDMs (LPS-primed) from wild type (WT) or Gpr109a deficient mice were treated with different concentrations of (S)-BHB and stimulated with CoCrMo particles. **F** Quantification of active Caspase-1 (P20) and active IL-1β (P17). n = 3. **G**  Representative image of Caspase-1 and IL-1β in supernatants by western blot. Results are mean ± SEM. *p < .05, ^#^p < .001

BHB is known as a signal molecule that can bind and activate Gpr109a (G protein-coupled receptor 109a) or act as a HDAC (histone deacetylase) inhibitor. We observed two HDAC inhibitors, TSA and LBH589, did not affect the activation of the inflammasome. Then we used a Gpr109a ligand agonist, niacin, to understand whether the inhibitory effect of BHB on macrophages was mediated by the receptor. The result showed that, unlike BHB, niacin had no effect on CoCrMo particles induced inflammasome activation (Fig. 4A and B). The analysis of IL-1β by ELISA also verified the results (Fig. 4C). BHB is a chiral compound, which consists of (R)-BHB and (S)-BHB [31]. We observed that the inhibitory effect of (R)-BHB was not altered in Gpr109a deficient or sufficient BMDMs (Fig. 4D and E). What’s more, (S)-BHB, the chiral compound enantiomer of (R)-BHB, which could not enter the TCA cycle, but had a high affinity for the Gpr109a receptor, had a similar inhibitory effect on the NLRP3 inflammasome independently of Gpr109a (Fig. 4F and G).

### BHB inhibit the differentiation and function of osteoclasts in vitro

**Fig. 5:**
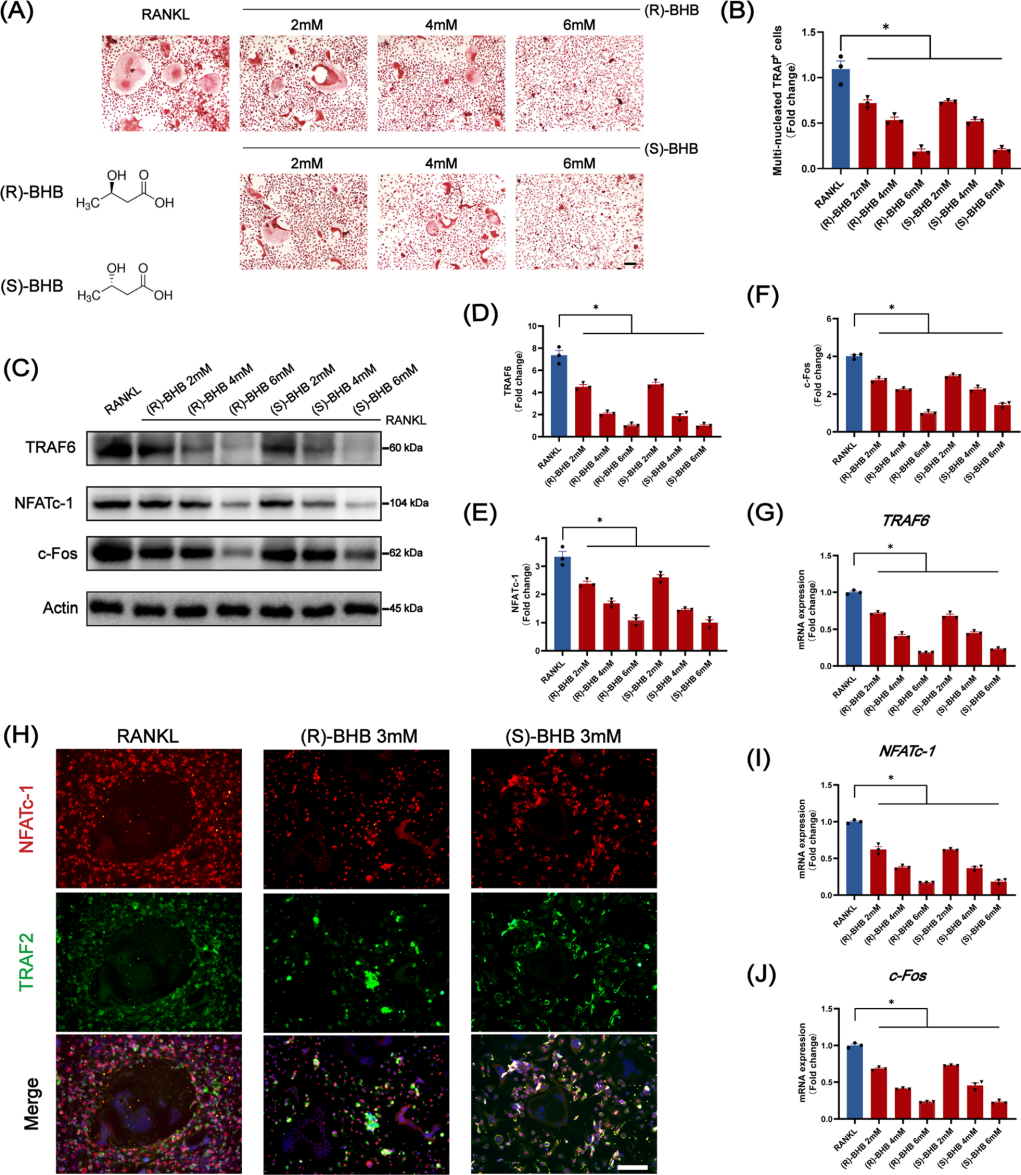
BHB inhibits the differentiation of osteoclast. **A** Representative TRAP staining of osteoclast. Scale bar: 200 μm. **B** Quantification of multi-nucleated TRAP-positive cells. **C** Representative image of TRAF6, NFATc-1, c-Fos after invention with BHB by western blot. **D**–**F** Quantification of TRAF6, NFATc-1, and c-Fos. n = 3. **G** The gene expression of TRAF6. n = 3. **H** Representative co-staining (NFATc-1 and TRAF2) immunofluorescence image of osteoclast. Scale bar: 100 μm. **I**–**J** The gene expression of NFATc-1 and c-Fos. Results are mean ± SEM. *p < .05

Given that butyrate, structurally related to BHB, had been reported to regulate the differentiation of osteoclast, thus we further investigated the effects of BHB on osteoclastogenesis. Then we used different doses of (R)-BHB and (S)-BHB during osteoclast differentiation to assess the effect of BHB on osteoclast differentiation in vitro. As Fig. 5A showed, no matter what kind dose of (R)-BHB or (S)-BHB revealed a suppressed ability on osteoclast differentiation with the fewer number of multi-nucleated TRAP-positive osteoclasts (Fig. 5B). Additionally, this inhibitory effect was dose-dependent according to our results. At an early time, BHB could inhibit the expression of TRAF6 and NFATc-1, two essential genes to osteoclastogenesis stimulated by receptor activator of nuclear factor-κB ligand (RANKL). And the expression of c-Fos was restricted to a low level from the immunoblotting result (Fig. 5C–F). These osteoclast differentiation-related genes were also suppressed by BHB intervention (Fig. 5G, I, J). Furthermore, the co-staining with TRAF 2 and NFATc-1 of osteoclast also confirmed the result (Fig. 5H).

**Fig. 6:**
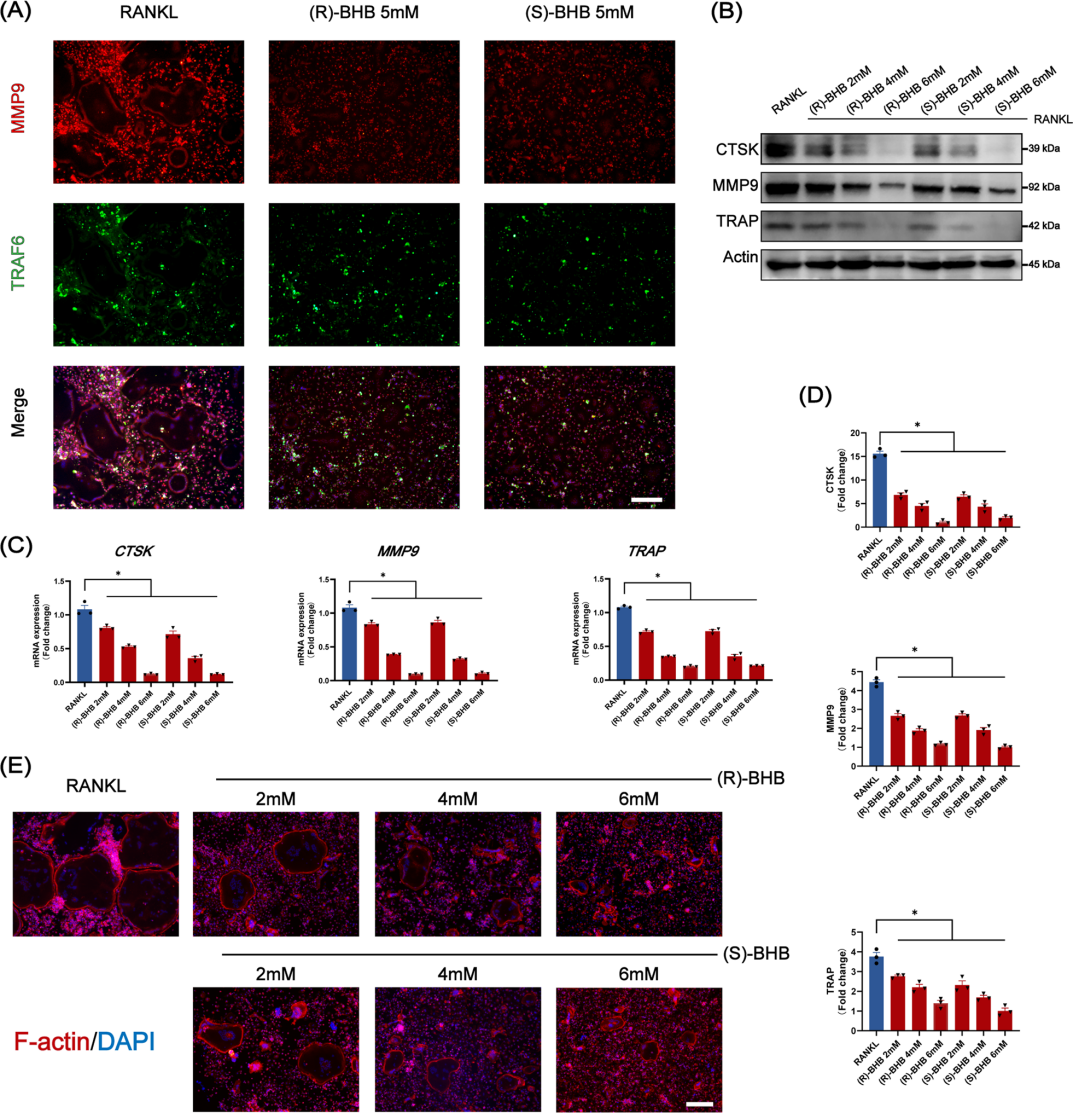
BHB inhibits that function of osteoclast. **A** Representative co-staining (MMP9 and TRAF6) immunofluorescence image of osteoclast. Scale bar: 200 μm. **B** Representative image of CTSK, MMP9, TRAP after invention with BHB by western blot. **C** The gene expression of CTSK, MMP9, and TRAP. n = 3. **D** Quantification of CTSK, MMP9, and TRAP. n = 3. **E** Representative Phalloidin labeling F-actin image of osteoclasts. Scale bar: 200 μm. Results are mean ± SEM. *p < .05

After confirming the inhibitory effects of BHB on osteoclast differentiation, we then investigated the effects of BHB on osteoclast function. As Fig. 6A–D showed, the expression of CSTK, TRAP, and MMP9, which were related to bone resorption and extracellular matrix degradation, was greatly diminished after incubation with R-BHB or S-BHB. Additionally, both R-BHB and S-BHB could reduce the formation of F-actin ring, which was contributed to bone resorption, indicating that BHB could affect the function of osteoclast in vitro (Fig. 6E).

Taken together, these results showed that both (R)-BHB and (S)-BHB could inhibit the differentiation and function of osteoclasts in vivo.

### Nutritional intervention with BHB alleviates osteolysis in vivo

**Fig. 7:**
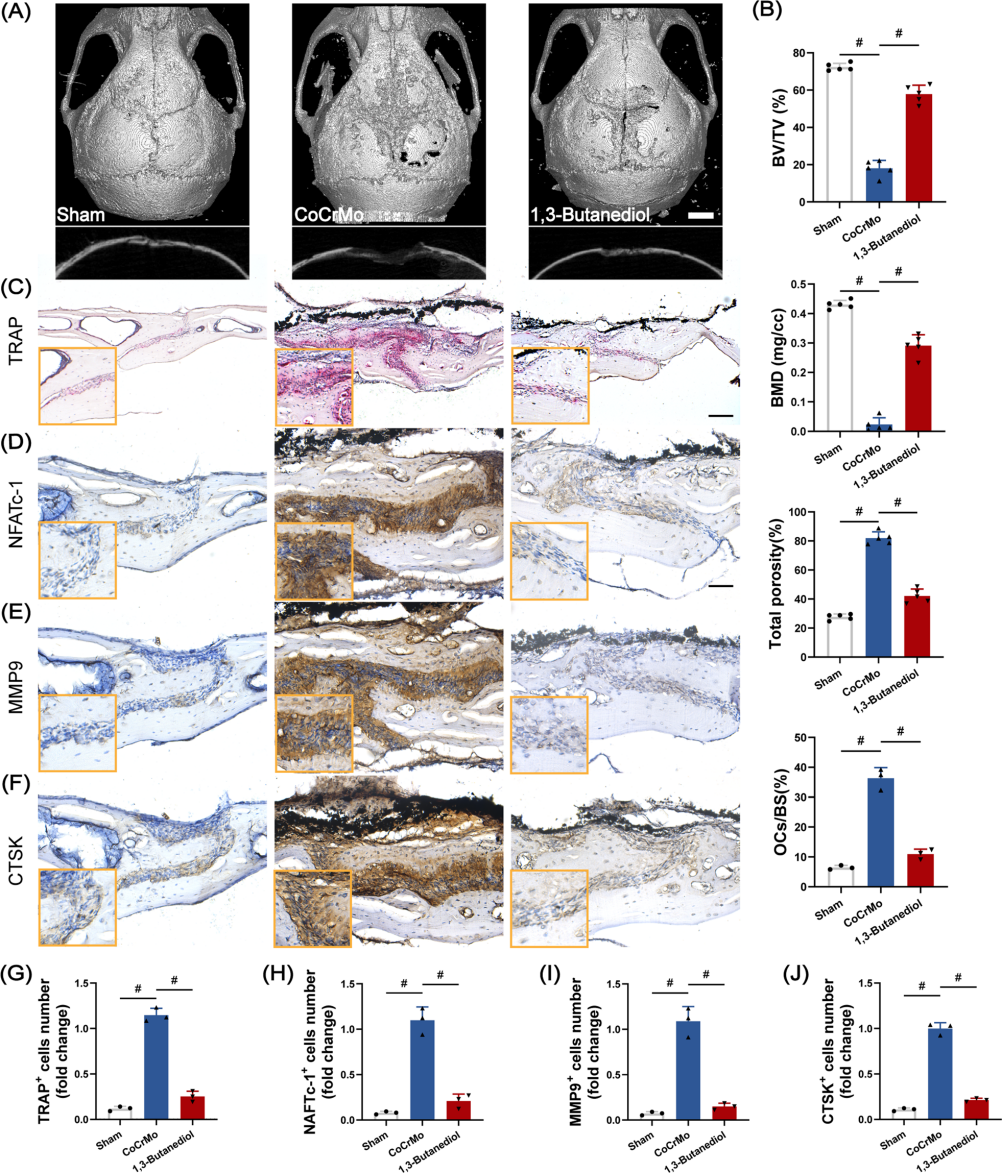
BHB alleviates osteolysis in vivo. **A** Representative image of Micro-CT 3D reconstruction of Calvarium. Scale bar: 5 mm. **B** Quantification of bone volume to tissue volume ratio (BV/TV), bone mineral density (BMD), total porosity, and the percentage of osteoclast surface per bone surface (OCs/BS). n = 5. **C** Representative image of TRAP staining. Scale bar: 200 μm. **D** Representative image of NFATc-1 immunohistochemical staining. Scale bar: 200 μm. **E** Representative image of MMP9 immunohistochemical staining. **F** Representative image of CTSK immunohistochemical staining. **G** Quantification of TRAP-positive cells. **H** Quantification of NFATc-1 positive cells. **I** Quantification of MMP9 positive cells. **J** Quantification of CTSK positive cells. Results are mean ± SEM. ^#^p < .001

Next, to verify the benefits of BHB in vivo, the mice were first subjected to osteolysis surgery. At the same time, the mice in 1, 3-butanediol group were provided with a drinking water containing 20% (v/v) 1, 3-butanediol (the BHB precursor). After two weeks of the intervention, mice were sacrificed for MicroCT analysis. We observed that a high level of circulating BHB was associated with the alleviation of osteolysis. Mice received with 1,3-butanediol reveal a high level of bone volume to tissue volume (BV/TV), bone mineral density (BMD), and a lower value of total porosity (Fig. 7A and B). Then we performed the TRAP and immunohistochemical staining with calvarial slices. As Fig. 7C showed, mice received 1,3-butanediol revealed fewer TRAP positive cells and a lower percentage of osteoclast surface per bone surface (OCs/BS, %) (Fig. 7B, C and G). Besides, the expression of osteoclast differentiation-related protein NFATc-1, bone resorption related protein CTSK, and extracellular matrix degradation-related protein MMP9 was obviously inhibited with the fewer number of positive cells (Fig. 7D–F, H–J). Collectively, BHB could also suppress the differentiation and function of osteoclasts in vivo.

### The effects of BHB on osteoclast may depend on inhibiting histone deacetylase

**Fig. 8:**
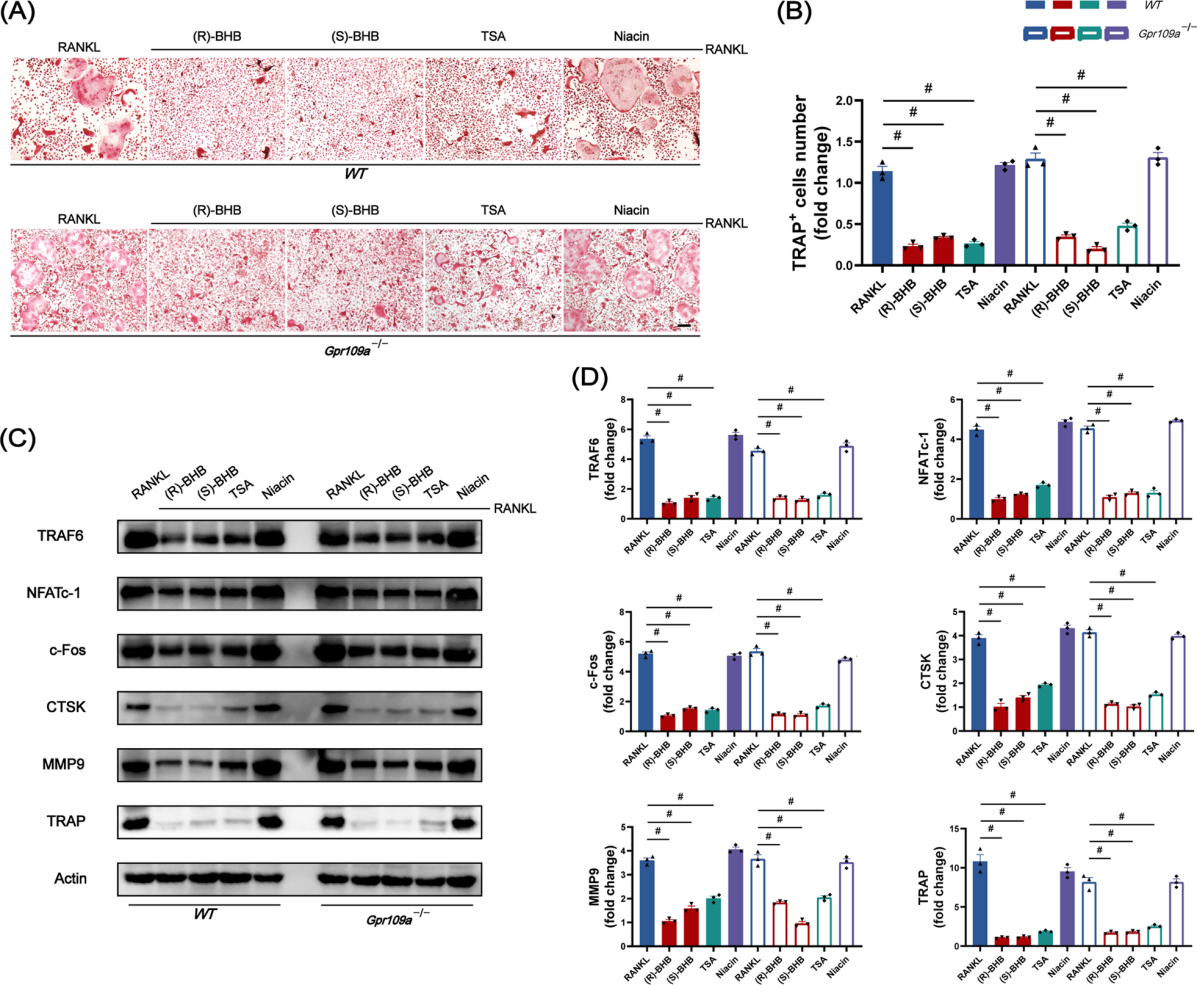
The inhibitory effects of BHB on osteoclast rely on HDAC suppression. **A** Representative TRAP staining of osteoclast. Scale bar: 200 μm. **B** Quantification of multi-nucleated TRAP-positive cells. **C** Representative image of TRAF6, NFATc-1, c-Fos, CTSK, MMP9, and TRAP after invention with BHB by western blot. **D** Quantification of TRAF6, NFATc-1, c-Fos, CTSK, MMP9, and TRAP. n = 3. Results are mean ± SEM. ^#^p < .001

As discussed above, BHB could activate the Gpr109a receptor or inhibit HDAC (histone deacetylase). We first measured the HDAC activity to confirm the inhibitory effect of BHB on histone deacetylase. As the result showed, BHB have a lasting suppressed effect on HDAC activity (Additional file 1: Fig. S4A). According to the previous study, BHB mainly affected class I HDACs (1,3) and class IIa HDACs (4) [36, 37]. To confirm the involvement of HDAC suppressed by BHB in osteoclast differentiation, we used selective short hairpin-mediated RNAs (shRNAs) to deplete HDAC3/4 in pre-osteolcasts. The selective shRNA for HDAC3/4 suppressed their cognate HDAC expression by at least 65% (Additional file 1: Fig. S4B-C). We observed that these pre-osteoclasts with HDAC 3/4 depletion showed fewer multi-nucleated TRAP positive osteoclasts during osteoclastogenesis, which is similar to the BHB intervention (Additional file 1: Fig. S4D-E). Furthermore, the inhibition of HDACs with non-selective histone deacetylase inhibitor TSA (Trichostatin A) also revealed a negative effect on the differentiation of osteoclasts (Fig. 8A and B). Therefore, the suppressed effects of BHB on osteoclasts may be via inhibiting HDAC (histone deacetylase) like its structurally related metabolite butyrate.

To verify the role of Gpr109a in the effects of BHB in osteoclasts, we used Niacin that had been reported to activate the Gpr109a receptor. We observed that, unlike TSA, Niacin did not affect the differentiation of osteoclasts (Fig. 8A and B). Additionally, the inhibitory effects of BHB did not alter in Gpr109a deficient osteoclast as it in Gpr109a sufficient osteoclast (Fig. 8A and B). We then detected osteoclast-related proteins and found that they were consistent with the trend of TRAP staining. Both (R)-BHB and (S)-BHB had an inhibitory effect on Gpr109a deficient osteoclast (Fig. 8C and D). It suggested that the Gpr109a receptor is not required for BHB to inhibit osteoclast differentiation. It may be mainly dependent on suppressing histone deacetylase.

**Fig. 9:**
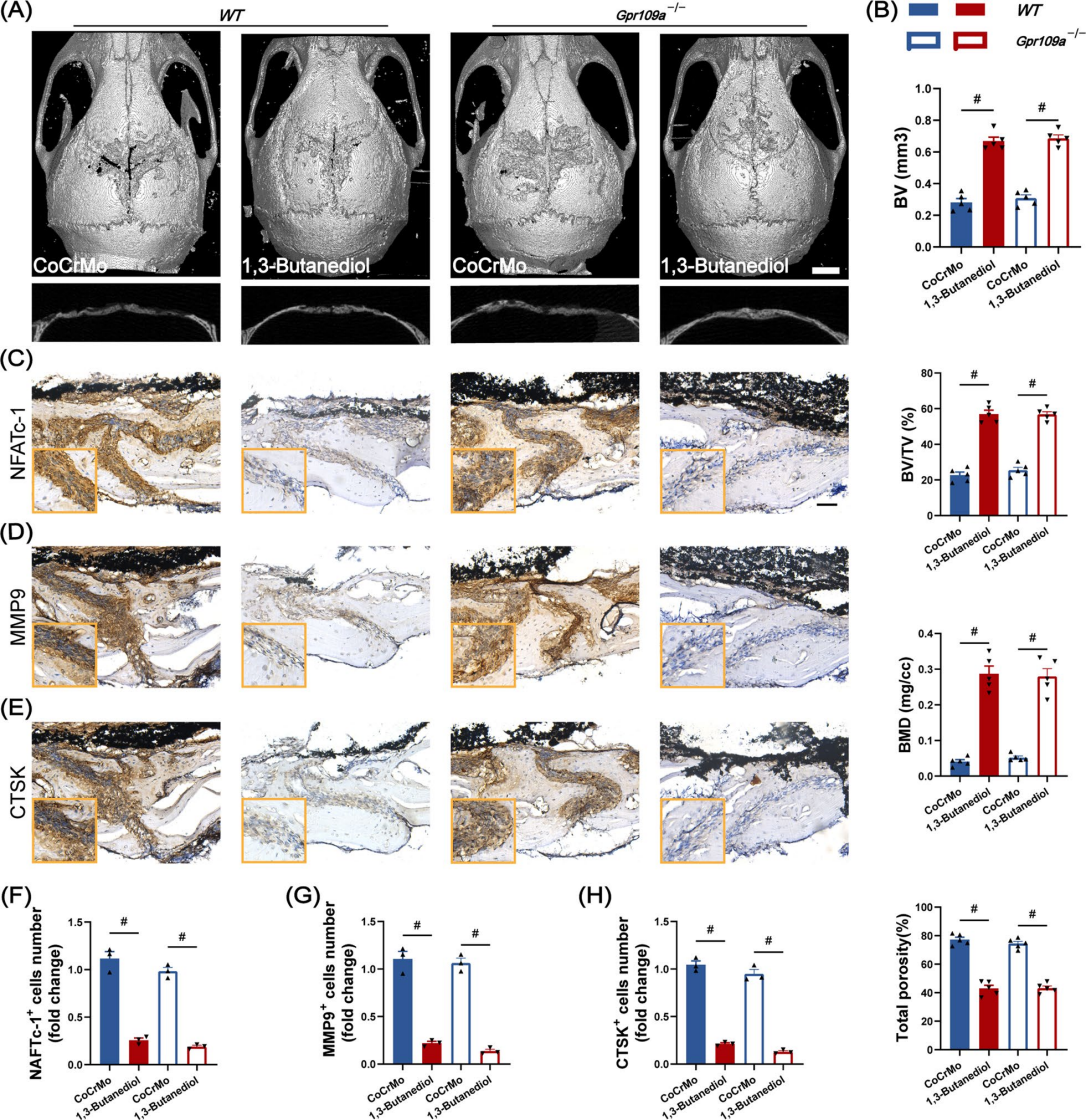
The inhibitory effect of BHB on osteoclast did not require the Gpr109a receptor. **A** Representative image of Micro-CT 3D reconstruction view of Calvarium. Scale bar: 5 mm. **B** Quantification of bone volume (BV), bone volume to tissue volume ratio (BV/TV), bone mineral density (BMD), and total porosity. n = 5. **C** Representative image of NFATc-1 immunohistochemical staining. Scale bar: 200 μm. **D** Representative image of MMP9 immunohistochemical staining. **E** Representative image of CTSK immunohistochemical staining. **F** Quantification of NFATc-1 positive cells. **G** Quantification of MMP9 positive cells. **H** Quantification of CTSK positive cells. Results are mean ± SEM. ^#^p < .001

To confirm the role of the Gpr109a receptor in vivo, we treated Gpr109a knockout or wild type mice with 1,3-butanediol. Deficient of Gpr109a receptor also did not affect the benefits of BHB on osteolysis. As Fig. 9A showed, the erosion pits were fewer and smaller with 1,3-butanediol intervention group compared to the operation alone group (CoCrMo) in wild type or Gpr109a knockout mice (Fig. 9A). The bone metabolism parameters of 1,3-butanediol intervention group in Gpr109a knockout mice were similar to its wild type littermates (Fig. 9B). Furthermore, calvarium slices from Gpr109a knockout mice with 1,3-butanediol intervention still revealed stable inhibitory effects of NFATc-1, MMP9, and CTSK expression (Fig. 9C–H).

Taken together, the regulation of BHB on osteoclasts may mainly rely on the suppression of histone deacetylase rather than Gpr109a receptor activation.

## Discussion

The development of implant failure was mainly attributed to the periprosthetic osteolysis caused by wear particles [38]. After total joint arthroplasty (TJA), wear particles from prosthesis were mechanically generated from the articulating surface. With the release of wear particles, many kinds of immune cells and inflammatory mediators would be involved in the immune response to these tiny particulates [2, 39, 40]. They were usually phagocytosed by the domain cells of monocytic lineage [2]. We and others have described the importance of the NLRP3 inflammasome activation in macrophages during osteolysis [3–7]. Mechanistically, Nlrp3 will be involved in the immune response to wear particles. Then Nlrp3 associates with the adopter protein ASC to form the complex, which in turn recruits the effector pro caspase-1 and cleaves it into the active form. Later, the active form caspase-1 (P20) could cleave IL-1β pro into the active form IL-1β (P17) [3–7]. At the same time, the active caspase-1 is qualified for GSDMD (gasdermin D) to generate GSDMD-NT (N-terminal fragment), which will in turn form pores in the membrane to release IL-1β, known as pyroptosis. IL-1β and other inflammatory cytokines will cause bone resorption by activating osteoclasts [3, 5–7, 16]. Although the understanding of the underlying mechanism of wear particles induced osteolysis has been substantially improved in recent years, there are still many challenges in effective and timely intervention to avoid the revision surgery, which will reduce the pain and economic burden of patients. The main barrier is to detect the early osteolysis, cause patients at this moment are often undiagnosed or asymptomatic [2]. If the effective intervention has been implemented timely in the early step of osteolysis, the possibility of developing aseptic loosening or even implant failure will undoubtedly be greatly reduced. However, it is obviously unrealistic to take drugs with side effects without a diagnosis. Thus, we investigated the effects of the ketone body β-hydroxybutyrate on osteolysis, which has been applied in multiple diseases.

In this work, we first examine the effect of ketone body β-hydroxybutyrate (BHB) and acetoacetate (AcAc) on the NLRP3 inflammasome activation triggered by CoCrMo particles. From our results, it is suggested that only β-hydroxybutyrate (BHB) could deactivate the NLRP3 inflammasome as well as pyroptosis activated by CoCrMo particles in ketone bodies. As an important metabolite in the ketogenic diet, β-hydroxybutyrate (BHB) has been reported to be with diverse functions. It is no longer considered as the alternative metabolic fuel resource, but a small molecule that can regulate immunity, genes expression, and even lifespan in mammals [17, 41]. For example, Sleiman et al. showed that ketone body β-hydroxybutyrate promotes the expression of brain-derived neurotrophic factor [42], while Rahman et al. found that the β-hydroxybutyrate receptor activated a neuroprotective subset of macrophages [30]. HCAR2 (hydroxycarboxylic acid receptor 2), known as Gpr109a, is considered to be a common mechanism of β-hydroxybutyrate action. Another underlying mechanism is the inhibition of histone deacetylase (HDAC) [17, 41]. Although our previous study showed that butyrate, a gut microbiota metabolite structurally related to BHB, relied on Gpr109a receptor to suppress the NLRP3 inflammasome activation upon titanium particles. Additionally, another study we're doing shows that niacin, a Gpr109a agonist, could also partly inhibit the inflammasome activation upon CoCrMo alloy particles in a high level, which is not consistent with the result of niacin in this study. It should be due to the concentration of niacin. However, the deactivation of BHB on inflammasome activation was not obviously changed in Gpr109a deficient BMDMs according to our result. At least, the most part of the inhibitory effects of BHB on inflammasome activation did not rely on Gpr109 receptor. Similarly, the result of the application of non-selective HDAC inhibitors revealed that the action of the inhibition of histone deacetylase did not significantly affect the activation of inflammasome upon CoCrMo alloy particles stimulation. In summary, our work revealed that the effect of β-hydroxybutyrate (BHB) on CoCrMo particles induced inflammasome activation did not require Gpr109a or depended on histone deacetylase suppression, which is similar to the previous study that β-hydroxybutyrate (BHB) deactivate the NLRP3 inflammasome activated by urate crystals, ATP and lipotoxic fatty acids independently of Gpr109a receptor and histone deacetylase (HDAC) suppression [31].

β-hydroxybutyrate (BHB) is structurally related to butyrate, which has been proven to be a regulator of bone metabolism [43]. Many works confirmed that butyrate had an effective inhibitory action on osteoclast differentiation and bone resorption, while Tyagi et al. reported that butyrate stimulated bone formation via T regulatory cell-mediated regulation of Wnt10B expression [44]. Given that osteoclasts activated by IL-1β and other inflammatory cytokines were attributed to the bone resorption in osteolysis, we next investigated the relationship between osteoclast and ketone body β-hydroxybutyrate (BHB). First, we performed the TRAP staining of osteoclast in vitro to figure out the effect of β-hydroxybutyrate (BHB). As our results showed, both (R)-β-hydroxybutyrate (R-BHB) and its chiral compound enantiomer (S)-β-hydroxybutyrate (S-BHB) had a dose-dependent inhibitory effect on osteoclast differentiation. Furthermore, the ketone body also impaired the function of bone resorption and extracellular matrix degradation of osteoclast. It is suggested that F-actin could be seen in podosomes of osteoclasts by Fluorescence-based staining [45]. These podosomes were arranged in dense podosome belts or actin rings (the sealing zone) [45]. The dynamic actin-rich ring structure would attache osteoclasts to the bone surface and bone resorption occurred in the space between the bone and osteoclasts defined by the sealing zone (known as the resorption lacuna), containing all proteolytic enzymes [46]. This kind of podosome arrangement is a hallmark of mature, resorbing osteoclasts. Apparently, these processes could be restrained by BHB via affecting the formation of actin rings and expression of TRAP, CTSK, and MMP9, which were responsible for the bone resorption. However, unlike inflammasome, we found that the inhibitory effects of ketone body BHB on osteolcast differentiation may mainly relied on histone deacetylase inhibition rather than Gpr109a activation. As the result showed, TSA, a non-selective HDAC inhibitor, which is commonly used for the research of HDAC inhibition [31, 43], did not affect the activation of inflammasome upon CoCrMo alloy particles. The mechanism of inflammatory osteolysis can normally be divided into two main steps: inflammation and osteoclast differentiation [47]. Then, we investigated the effect of BHB during the differentiation of osteoclasts. It is suggested that HADC activity was critical for osteoclast differentiation as well as its function. When it was inhibited by TSA or BHB, the step of osteoclastogenesis was greatly diminished. Fewer multi-nucleated TRAP positive osteoclasts could be seen after TSA or BHB intervention. Moreover, the formation of F-acting ring was also impaired, which was responsible for bone resorption. More importantly, HDAC 3/4 depletion by sh-RNA in pre-osteoclasts revealed a similar negative effects on osteolcasts differentiation. Therefore, it seems that the inhibitory effect of BHB on HDAC activity mainly affect the later step of osteolysis, osteoclast differentiation. The inhibition of HDAC activity by BHB was critical for osteolysis and not for inflammsome inhibition, since it was mainly involved in osteoclasts differentiation rather than inflammasome deactivation. Although our work did not focus on the action of β-hydroxybutyrate on osteoblast, it was consensus that the osteoblast is not the dominant cell during osteolysis. The inflammation response and the subsequent abnormal activation of osteoclasts are mainly attributed to the aseptic loosening followed by periprosthetic osteolysis [2, 39, 48].

**Fig. 10:**
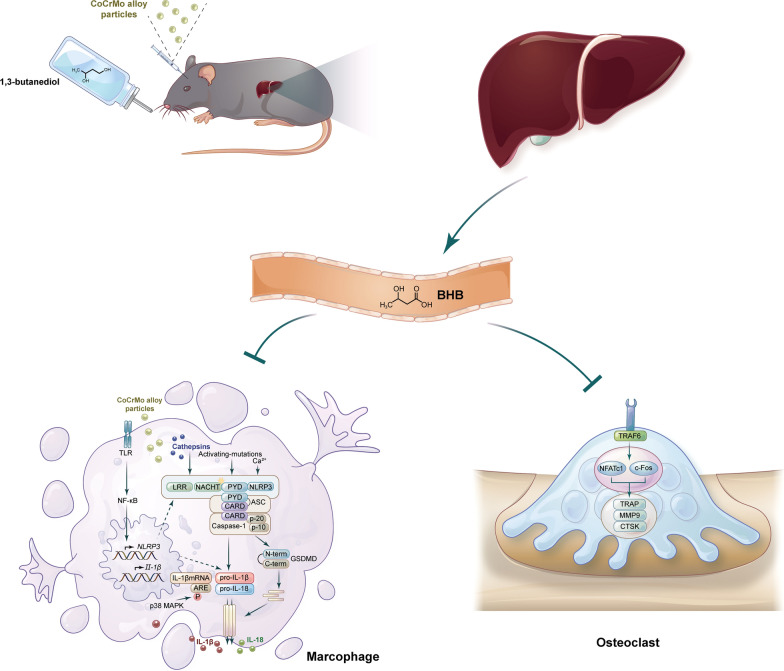
The ketone body β-hydroxybutyrate (BHB) alleviated CoCrMo alloy particles induced osteolysis via inhibiting the NLRP3 inflammasome activation and osteoclasts differentiation

In conclusion, our work suggested that the ketone body β-hydroxybutyrate (BHB) could deactivate the activation of NLRP3 inflammasome as well as osteoclasts, which were considered as a common mechanism during osteolysis (Fig. 10). Moreover, considering that the ketone body may also be associated with osteoblasts, the benefits of β-hydroxybutyrate on bone metabolism should be investigated as its structurally related molecule, butyrate.

## Methods

### Animal study

Nine to ten weeks of C57BL/6 J male mice were bought from Suzhou Healthytech Bio-pharmaceutical Co., Ltd. (Suzhou, China). Mice were free to food and water in a specific-pathogen-free (SPF) environment with a standard of 12 h light and 12 h dark cycle at 22 ± 1.5 °C. After 1–2 weeks of accommodation, mice were randomly assigned to five groups and subjected to osteolysis surgery. The osteolysis surgery was performed as previously described [4]. Briefly, CoCrMo alloy particles were subjected to incubation under 180 °C for 8 h and resolved in ethanol solution for 1 day to remove endotoxins. The next day, CoCrMo alloy particles were resuspended in a concentration of 500 mg/ml with sterile PBS. After preparation of CoCrMo alloy particles, every mouse received 40 μl CoCrMo alloy particles solution through the 10 mm midline sagittal incision on calvarium. Each group received a chow diet. Mice in the 1,3-butanediol intervention group were treated with 20% (v/v) 1, 3-butanediol (sigma) in drinking water for ketone body research in vivo as in previous study [28].

### Cell culture

THP-1 macrophage: THP-1 cells were purchased from Procell (Wuhan, China). THP-1 cells were cultured in RPMI 1640 medium (Procell) with 10% fetal bovine serum and 1% antibiotic. After several weeks of proliferation, THP-1 were seeded to six-well plates. Next, THP-1 cells were differentiated to macrophages by 3 h incubation with 100 nM PMA (MedChemExpress, USA) and primed with LPS (Sigma, USA) for 3 h. Then sterile CoCrMo alloy particles solution was added to the medium. The final concentration of CoCrMo alloy particles in the medium was 0.1 mg/ml. Cells were incubated with different doses of acetoacetate (AcAc) (MedChemExpress, USA), β-hydroxybutyrate (BHB) (MedChemExpress, USA), or acetoacetate + β-hydroxybutyrate (AcAc + BHB) at the same moment of CoCrMo alloy particles intervention. Supernatants were collected for the analysis of ELISA and Western Blot.

Bone marrow derived macrophages (BMDMs): 9–10 weeks of mice were sacrificed in a sterile environment. Then tibial and femur were separated and bone marrow was collected by flushing with complete RPMI 1640 medium. Then cells were subjected to a red blood cell lysis buffer to remove the erythrocyte. Cell were resuspended after washing with sterile PBS for one time. The next day, the non-adherent cells were collected and seeded into six-well plates with complete RPMI 1640 medium containing 40 ng/ml M-CSF (R&D system, USA). The medium was changed every two days. After five to seven days of differentiation, cells were subjected to further study. For the activation of the NLRP3 inflammasome, cells were primed with LPS (Sigma, USA) for 3 h and then stimulated with CoCrMo particles, acetoacetate (AcAc), β-hydroxybutyrate (BHB), (R)-hydroxybutyrate (MedChemExpress, USA), (S)-hydroxybutyrate (MedChemExpress, USA), Niacin (MedChemExpress, USA), Trichostatin A (TSA) (MedChemExpress, USA), and LBH598 (Sigma, USA) for 6 h. The Supernatants and cell lysates were collected for western blot or ELISA analysis.

Osteoclast: bone marrow cells were collected from the tibial and femur of mice and cultured in α-MEM media overnight. Non-adherent cells were collected and resuspended with α-MEM medium containing 40 ng/ml M-CSF (R&D system, USA) on the next day. After three days of differentiation, RANKL (R&D system, USA) was added to medium with a concentration of 50 ng/ml. About 5–6 days, mature osteoclasts were subjected to further study.

### The characteristic of CoCrMo alloy particles

The image of SEM and CoCrMo alloy particles size distribution was performed by CeshiGo. Co., Ltd. (Nanjing, China).

### ASC oligomerization and speck formation

The details were provided in Additional file 1.

### HDAC3/4 knockdown in preosteoclast

Mouse HDAC3-shRNA and HDAC4-shRNA plasmids (Santa Cruz Biotechnology, Dallas, Texas) were used to knockdown HDAC3/4 genes in pre-osteoclasts (day 1) according to the manufacturer’s protocol. Control shRNA plasmids were used as a negative control. The percent of HDAC3/4 gene knockdown was assayed by western blot.

### MciroCT

The details were provided in Additional file 1.

### Western bolt

BMDMs were lysed by a cold buffer containing RIPA buffer (beyotime biotechnology, China), phosphatase, and proteinase inhibitors for 15 min. Then, the samples were centrifuged at 12,000*g* 5 min. The supernatants were subjected to a BCA assay (Beyotime Biotechnology, China) to determine the concentration of protein. The supernatant proteins were precipitated. The Western blot analysis was performed as previously described. The primary antibody of Caspase-1 (Cell Signaling Technology), IL-1β (Santa Cruz Biotechnology), Actin (Beyotime biotechnology), GSDMD (Abcam), TRAF6 (Proteintech), NFATc-1 (Abcam), TRAP (Abcam), CTSK (Santa Cruz Biotechnology), MMP9 (proteintech), c-Fos (proteintech). The quantification analysis was performed by ImageJ.

### HDAC activity

Osteoclasts incubated with or with out BHB were collected and lysed in a cold RIPA buffer (Beyotime biotechnology, China) containing a protease inhibitor (Beyotime biotechnology, China). The concentration of protein was determined by a BCA assay kit (Beyotime Biotechnology, China). Then, HDAC activity was measured by an HDAC activity assay kit (Amplite fluorimetric, USA) according to the supplier’s instructions.

### Enzyme-linked immunosorbent assay

The enzyme-linked immunosorbent assay of IL-1β and IL-18 in supernanants were performed with the ELISA kit (MultiSciences, China) according to the manufacturer’s instruction.

### qRT-PCR

The details were provided in Additional file 1.

### Immunohistochemistry, immunofluorescence staining and TRAP staining

The TRAP staining of mature osteoclast and calvarium slices was performed with a tartrate-resistant acid phosphatase kit (BZ Biotechnology, China) according to the manufacturer’s instruction.

For immunohistochemistry, 6 µm slices of calvarium were prepared and the immunohistochemistry staining was performed as previously described [4]. For immunofluorescence, differentiated osteoclasts were fixed by 4% paraformaldehyde and then performed staining. The primary antibodies of TRAF6 (Proteintech), TRAF2 (Proteintech), NFATc-1 (Abcam), TRAP (Abcam), MMP9 (Proteintech) were used.

Calcein-AM/Propidium Iodide (PI) Staining: After different stimulation, cells were washed twice with PBS. Then, cells were washed twice with 1× assay buffer. After that, cells were stained with 2 μM calcein-AM and 4.5 μM PI per well at 37 °C for 30 min. The images of the cells were acquired immediately by using a fluorescence microscope.

### Statistical analysis

A one-way analysis of variancepost (ANOVA) (post hoc Tukey) test within Sigmaplot 12.5 software (Systat Software, San Jose, CA, USA) was used for statistics. Data are expressed as mean ± SEM. p < 0.05 was considered statistically significant.

## Supplementary Information

**Additional file 1: Table S1.** Primer sequences used in qRT-PCR analysis. **Figure S1.** BHB inhibit NLPR3 inflammasome activated by CoCrMo alloy particles in THP-1 (PMA-induced) macrophages. **Figure S2.** BHB inhibit inflammasome activation induced cell death by Calcein/PI staining. **Figure S3.** BHB inhibits pyroptosis-induced LDH release. **Figure S4.** HDAC activity was involved in the differentiation of osteoclast.

## Data Availability

The datasets generated and/or analysed during the current study are not publicly available but are available from the corresponding author on reasonable request.
