# Supplementary material for: The ketone body β-hydroxybutyrate alleviates CoCrMo alloy particles induced osteolysis by regulating NLRP3 inflammasome and osteoclast differentiation
Source: J Nanobiotechnology. 2022 Mar 9;20:120. doi: 10.1186/s12951-022-01320-0 (PMC8905851; doi:10.1186/s12951-022-01320-0)
Supplement: Supplementary file 1 — Additional file 1: Table S1. Primer sequences used in qRT-PCR analysis. Figure S1. BHB inhibit NLPR3 inflammasome activated by CoCrMo alloy particles in THP-1 (PMA-induced) macrophages. Figure S2. BHB inhibit inflammasome activation induced cell death by Calcein/PI staining. Figure S3. BHB inhibits pyroptosis-induced LDH release. Figure S4. HDAC activity was involved in the differentiation of osteoclast. [file 12951_2022_1320_MOESM1_ESM.docx]

**Additional file 1**

**The ketone body β-hydroxybutyrate** **alleviates CoCrMo alloy particles induced osteolysis by regulating NLRP3 inflammasome and osteoclast differentiation**

Running title: β-hydroxybutyrate inhibits the NLRP3 inflammasome activation and osteoclast differentiation

Yanglin Wu^1 2#^, Yun Teng^1#^, Chenhui Zhang^1^, Ying Pan^3^, Qin Zhang^1^, Xu Zhu^1^, Naicheng Liu^1^ , Xinlin Su^1^, Jun Lin^1*^

^1^ Department of Orthopaedics, The First Affiliated Hospital of Soochow University, Soochow University, Suzhou 215006, China

^2^ Orthopaedic Institute, Medical College, Soochow University, Suzhou 215007, China

^3^ Department of Infectious Diseases, The Second Affiliated Hospital, Zhejiang University School of Medicine, Hangzhou, China

^#^ These authors contributed equally.

**Corresponding Authors:**

**^*^** Jun Lin, M.D., Ph.D., Department of Orthopaedics, The First Affiliated Hospital of Soochow University, No. 188 Shizi Street, Suzhou 215006, Jiangsu, China. Telephone: +86-512-67972165; Email: [linjun@suda.edu.cn](mailto:linjun@suda.edu.cn)

1. **Experimental procedures**

**1.1 ASC oligomerization and speck formation**﻿

LPS primed macrophages were stimulated with CoCrMo alloy particles in presence of β-hydroxybutyrate (BHB) for 6h. Then, the supernanants were changed with a cold buffer (0.5% Triton X-100, 50 mM Tris-HCl, PH 7.6, a protease inhibitor cocktail and 0.1 mM PMSF). After 30 min, the cell lysates were scraped and centrifuged at 3000 g for 10 min at 4°C. The pellets were washed twice with PBS and resuspended in 2mM DSS for 30 min. After incubation, the cross-linked pellets were subjected to Western Blot analysis.

To the observation of ASC speck, BMDMs were seeded into 24-well plate overnight. On the next day, the indicated regents were used to activate the inflammasome in macrophages. Next, BMDMs were fixed with 4% paraformaldehyde for 10 min and then performed ASC staining.

**1.2 MciroCT**

The calvarium was collected and scanned by micro-computed tomography (Micro-CT) (SkyScan 1176, Aartselaar, Belgium). The parameters were set with the current of 500 µA and the voltage of 50 kV. For quantification, the CT analyzer (SkyScan) was used. The scanning layer was 9 µm. A 3 mm diameter round of region of interest (ROI) containing 10 layers on every calvarium was selected for the analysis of bone metabolism: bone mineral density (BMD, mg/cc), the ratio of bone volume to tissue volume (BV/TV, %), and total porosity (%).

**1.3 qRT-PCR**

Total RNA was extracted from osteoclasts with a Simply P Total RNA Extraction Kit **(**BioFlux) according to the manufacturer’s instruction. The synthesis of cDNA and Q-PCR were performed as preciously described. The primer used was listed in supplementary table 1.

| Gene | Forward | Reverse |
| --- | --- | --- |
| *TRAF6* | 5´AAAGCGAGAGATTCTTTCCCTG3´ | 5´ACTGGGGACAATTCACTAGAGC3´ |
| *NFATc-1* | 5´GGTGCCTTTTGCGAGCAGTATC3´ | 5´CGTATGGACCAGAATGTGACGG3´ |
| Trap | 5´CGACCATTGTTAGCCACATACG3´ | 5´TCGTCCTGAAGATACTGCAGGTT3´ |
| *Ctsk* | 5´AGGGCCAACTCAAGAAGAAAACT3´ | 5´TGCCATAGCCCACCACCAACACT3´ |
| *Mmp9* | 5´GCTGACTACGATAAGGACGGCA3´ | 5´ TAGTGGTGCAGGCAGAGTAGGA3´ |
|  |  |  |

**Table S1.** Primer sequences used in qRT-PCR analysis.

**Abbreviation**

**
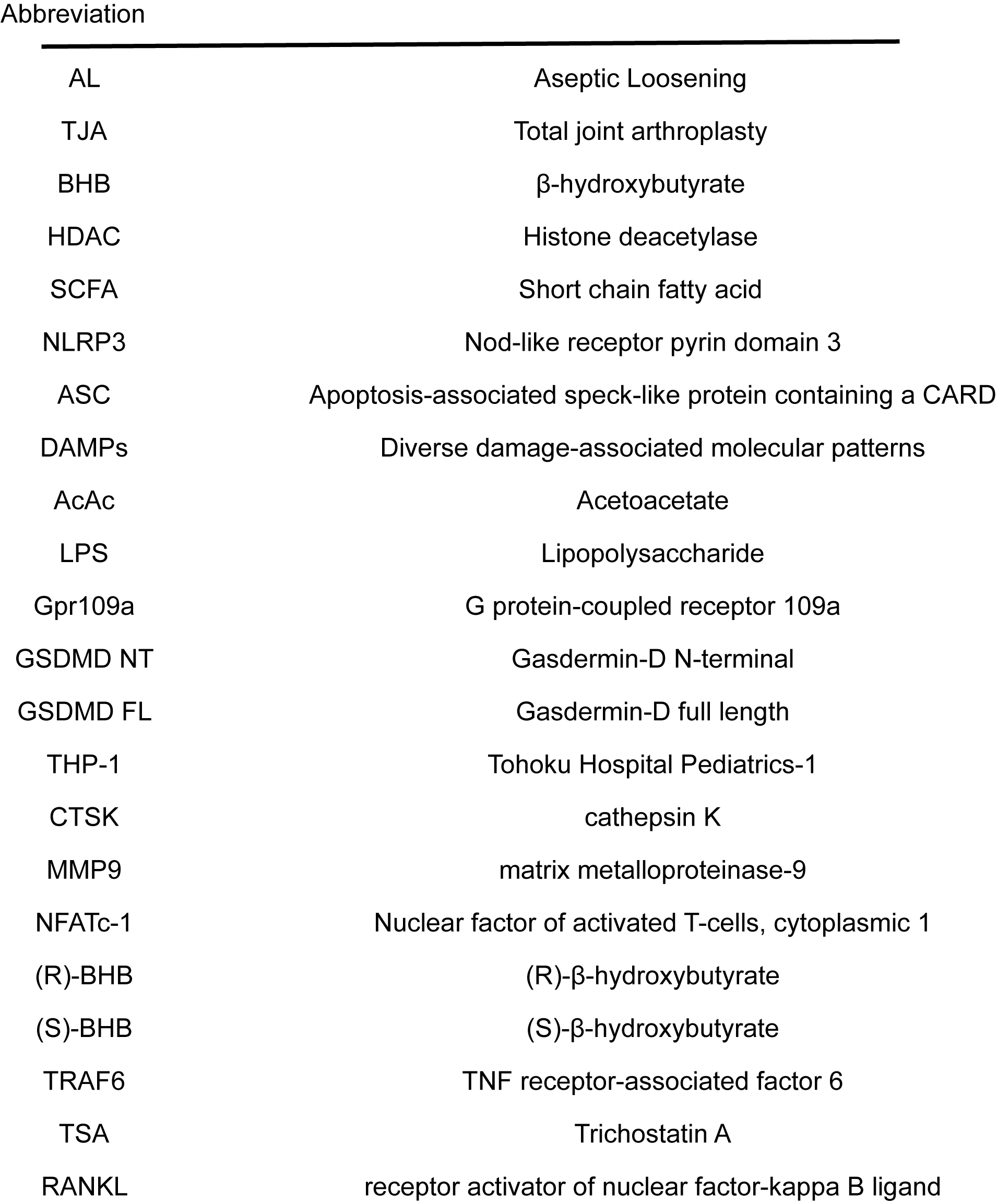
**

**
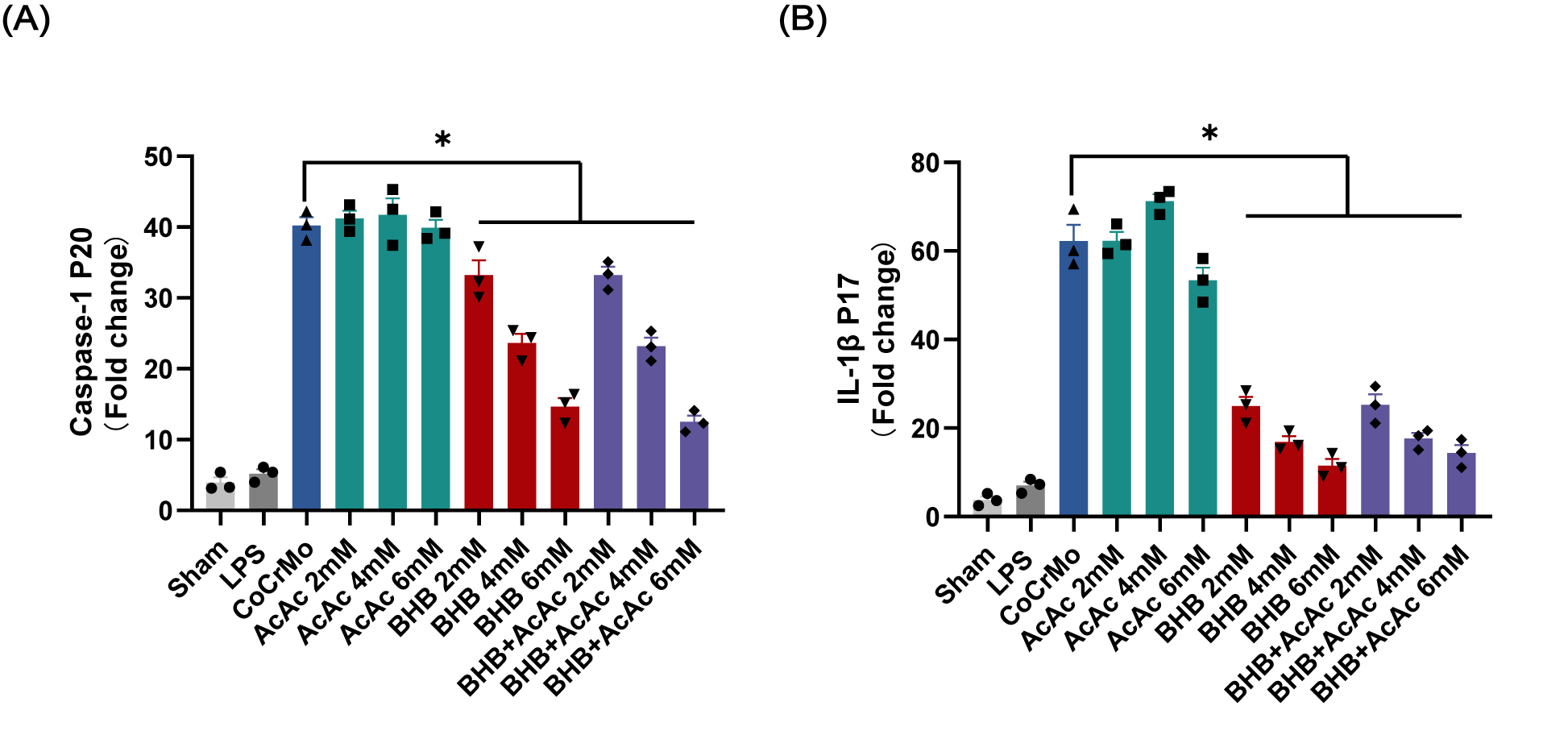
**

**Figure S1.** BHB inhibit NLPR3 inflammasome activated by CoCrMo alloy particles in THP-1(PMA-induced) macrophages. **(A)** Quantitation of active Caspase-1 p20 and **(B)** active IL-1β p17 band intensity as fold change. Results are mean ± SEM *** p < .001.

**
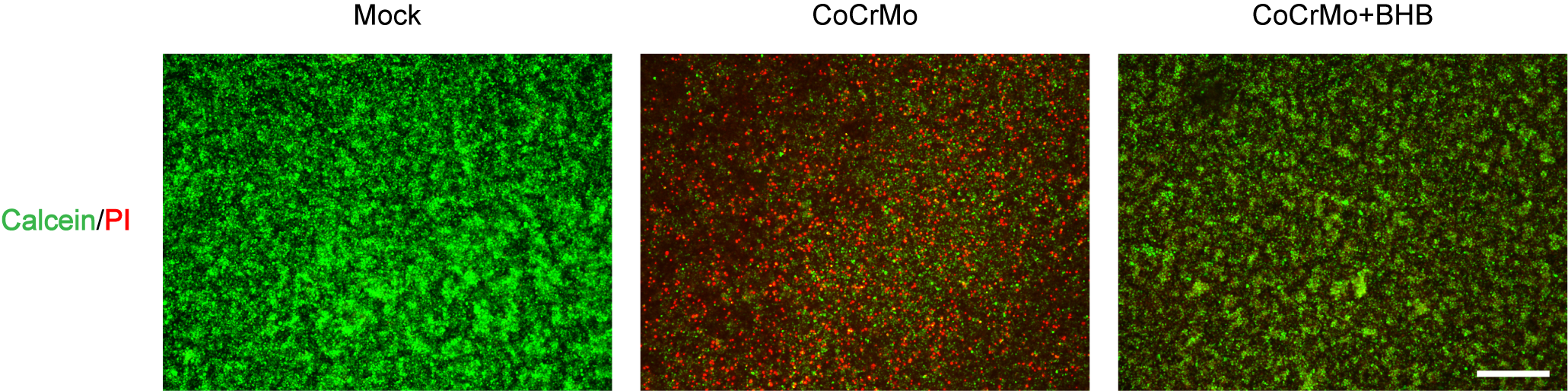
 Figure S2.** BHB inhibit inflammasome activation induced cell death by Calcein/PI staining. Scale bar: 100 μm.


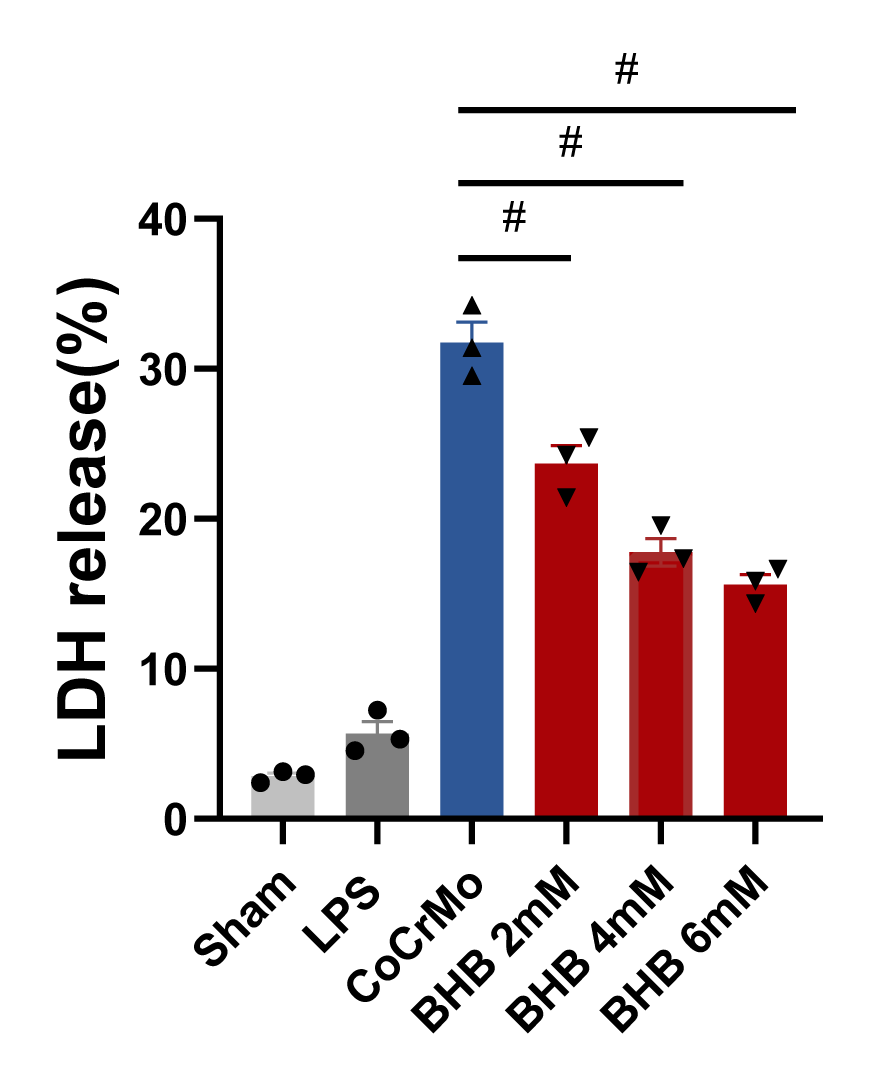


**Figure S3.** BHB inhibits pyroptosis-induced LDH release.


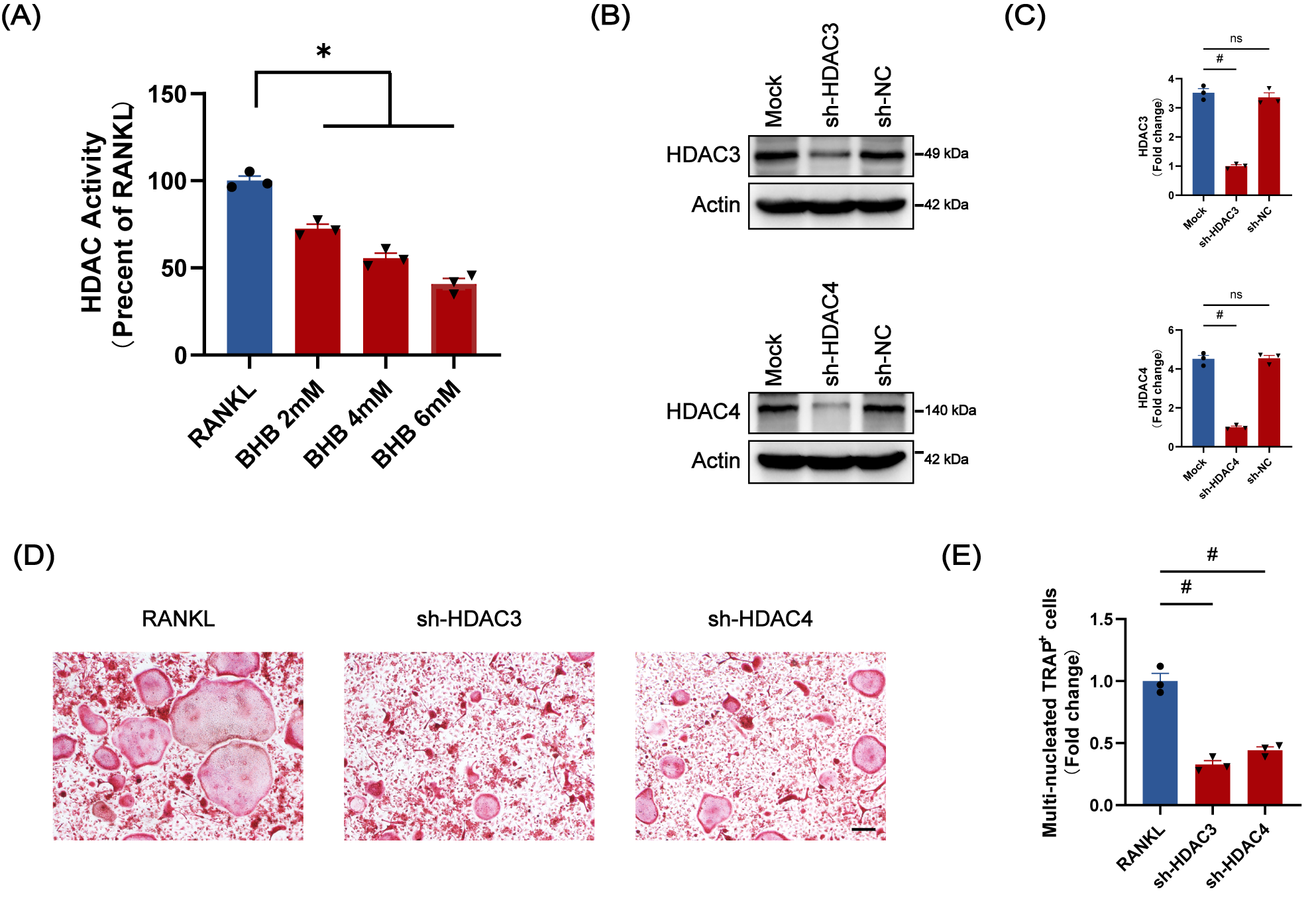


**Figure S4.** HDAC activity was involved in the differentiation of osteoclast. (A) BHB inhibited HDAC activity in osteoclasts. (B) Representative western blot image of HDAC3, HDAC4, and actin in pre-osteoclasts. (C) Quantification of HDAC3 and HDAC4. (D) Representative TRAP staining images of osteoclasts. Scale bar： 200μm. (E) Quantification of multi-nucleated TRAP-positive cells.
